# Supplementary material for: Synergistic Drug Combinations Prevent Resistance in ALK+ Anaplastic Large Cell Lymphoma
Source: Cancers (Basel). 2021 Sep 1;13(17):4422. doi: 10.3390/cancers13174422 (PMC8431561; doi:10.3390/cancers13174422)
Supplement: Supplementary file 1 [file cancers-13-04422-s001.zip › cancers-1318949-supplementary.pdf]

cancers

Supplementary Materials: Synergistic Drug Combinations Prevent Resistance in ALK+ Anaplastic Large Cell Lymphoma

Giulia Arosio, Geeta G. Sharma, Matteo Villa, Mario Mauri, Ilaria Crespiatico, Diletta Fontana, Chiara Manfroni, Cristina Mastini, Marina Zappa, Vera Magistroni, Monica Ceccon, Sara Redaelli, Luca Massimino, Anna Garbin, Federica Lovisa, Lara Mussolin, Rocco Piazza, Carlo Gambacorti-Passerini and Luca Mologni

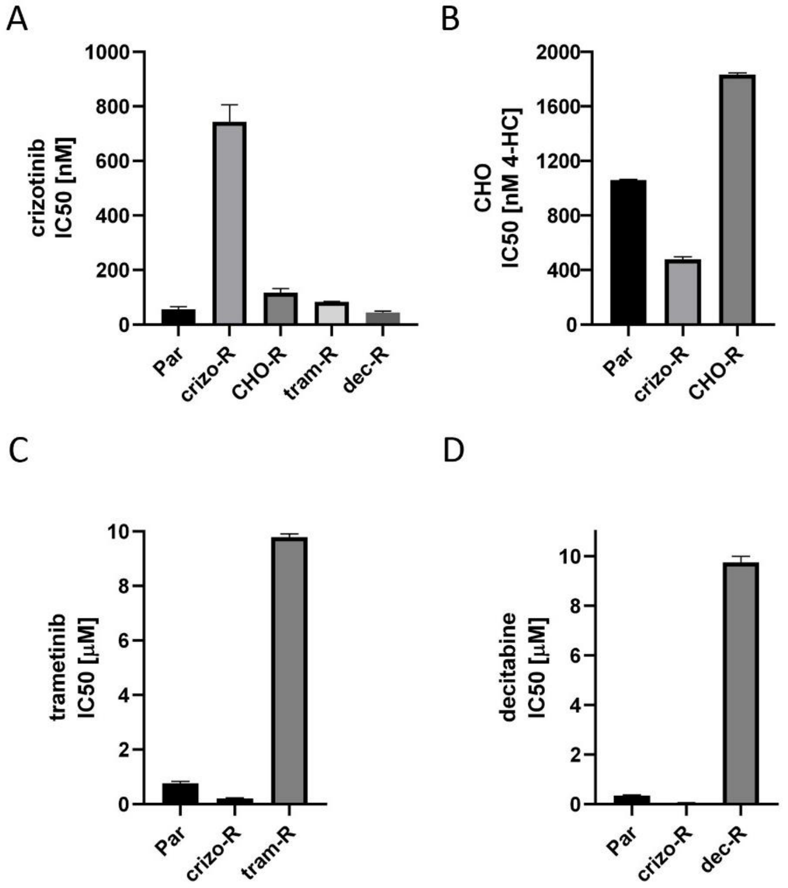

Figure S1. Cross-sensitivity of drug-resistant cells. SUP-M2 cells resistant (-R) to crizotinib, CHO, decitabine or trametinib were challenged with (**A**) crizotinib, (**B**) CHO, (**C**) trametinib, and (**D**) decitabine. The IC50 values (mean + SEM) are reported in the graphs. Par = parental cells.

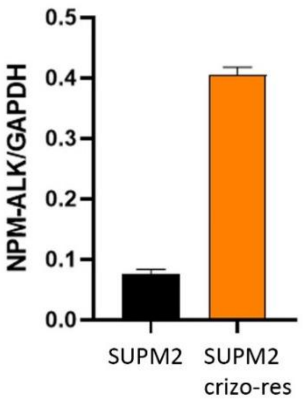

Figure S2. Quantitative analysis of NPM-ALK mRNA expression in parental (black bar) versus crizotinib-resistant SUP-M2 cells (orange).

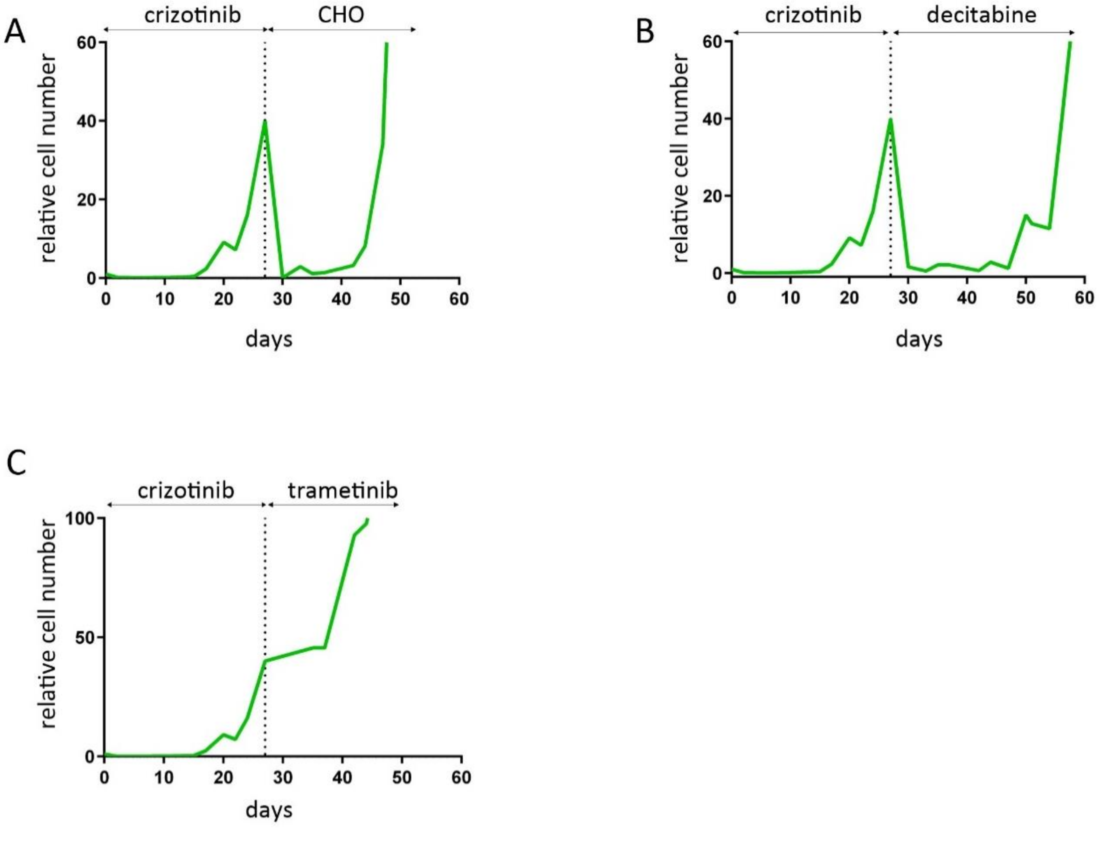

Figure S3. Sequential treatment combinations. SUP-M2 cells were grown in the presence of 100 nM crizotinib until a resistant population emerged. On day 27 of culture (dotted line) crizotinib-resistant cells were shifted to (**A**) CHO, or (**B**) decitabine, or (**C**) trametinib. In all three cases, a new double-resistant cell culture appeared. In the same experiment, simultaneous combinations confirmed full suppression of resistance, as depicted in Figure 1 (not shown here).

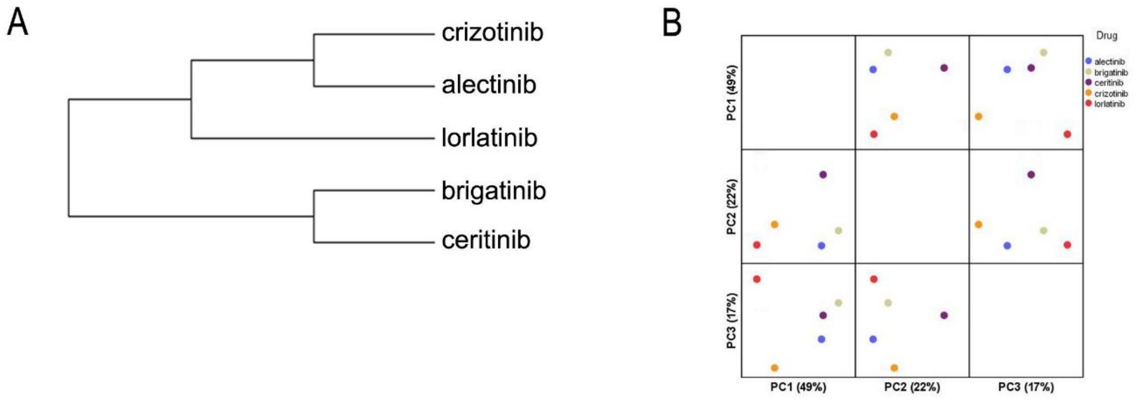

C

genotype	crizotinib	ceritinib	alectinib	ASP3026	brigatinib	PF-06463922
Q1064R	8	21	84	nd	307	782
L1122V	10	15	8	5	11	nd
G1128S	4	4	0	14	1	9
P1139S	4	6	1	2	2	nd
L1152R	4	11	2	37	0	3
C1156Y	10	3	2	4	2	2
C1156F	3	8	8	17	16	131
I1171N	8	7	16	7	4	8
I1171T	3	3	14	4	6	124
F1174I	2	0	0	2	1	2
F1174V	4	2	6	4	2	nd
F1174L	8	6	5	5	4	5
N1178H	1	2	0	2	2	2
L1196M	19	2	3	7	3	4
L1196Q	7	0	nd	nd	nd	nd
L1198F	0	36	18	5	8	nd
G1202R	26	11	29	16	17	56
S1206Y	15	1	1	1	2	nd
S1206C	17	7	9	16	19	nd
E1210K	3	7	6	10	12	48
G1269A	16	1	11	5	1	100
M1328I	2	2	3	nd	5	9

Figure S4. (**A**) Neighbour joining tree analysis shows clustering of ALK inhibitors based on their profiles of activity against mutants. Pairwise distances were calculated by Principal Component Analysis (**B**) of the data shown in panel C; the tree was inferred using the Minimum Evolution method. (**C**) Relative resistance index values calculated as mutant IC50 fold increase relative to wild-type ALK (WT = 1). The raw data were obtained using BaF3-NPM/ALK cells as described (ref. 11).

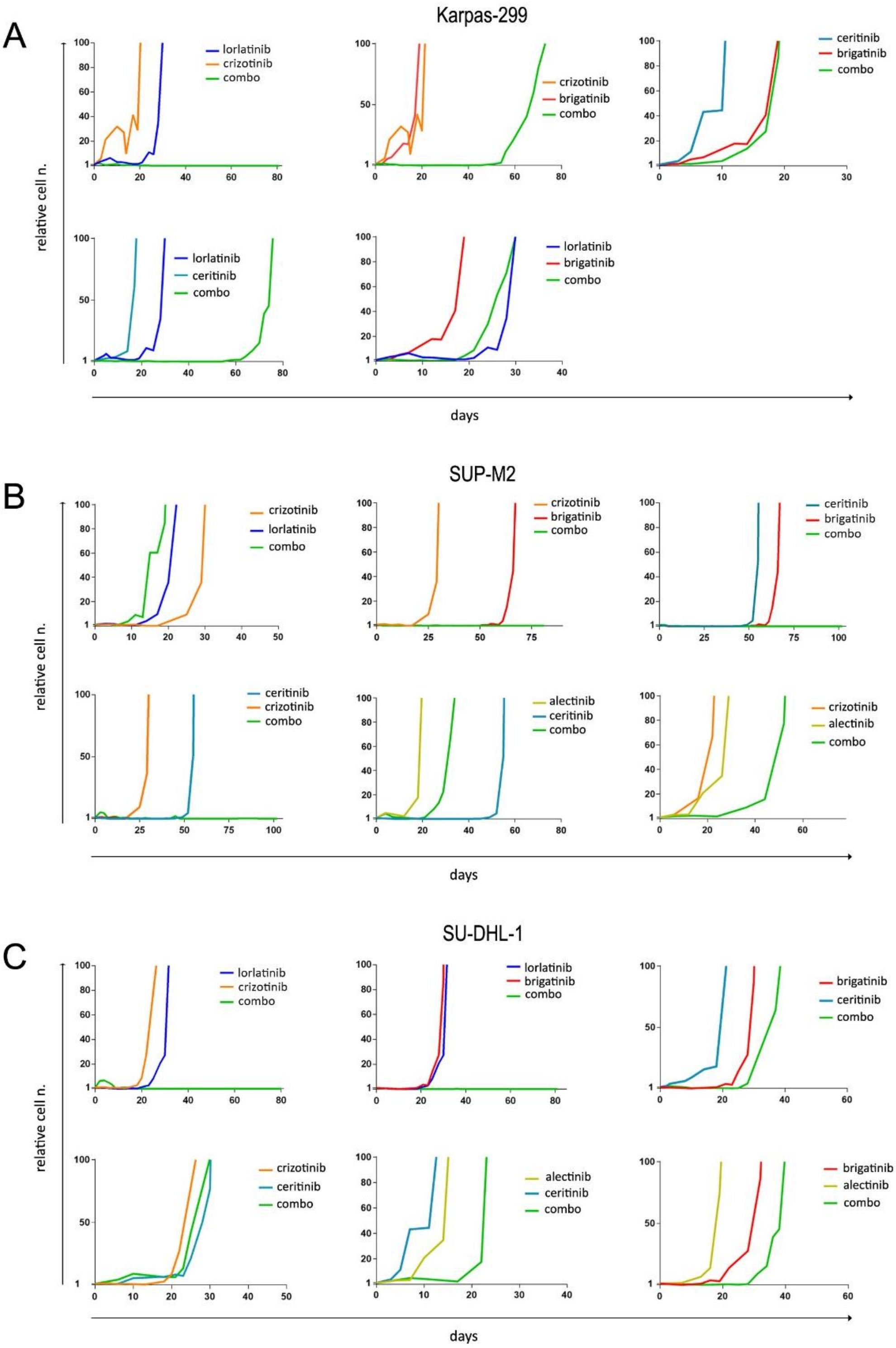

Figure S5. Long-term TKI-TKI combinations. (**A**) Karpas-299, (**B**) SUP-M2 and (**C**) SU-DHL-1, cells were cultured in the presence of the indicated inhibitors, full-dose single (crizotinib, 100 nM; brigatinib, 40 nM; ceritinib, 50 nM; lorlatinib, 8 nM) or half-dose combination. Cumulative cell count is shown, relative to day 0. Experiments were halted earlier when combo showed cells outgrowth.

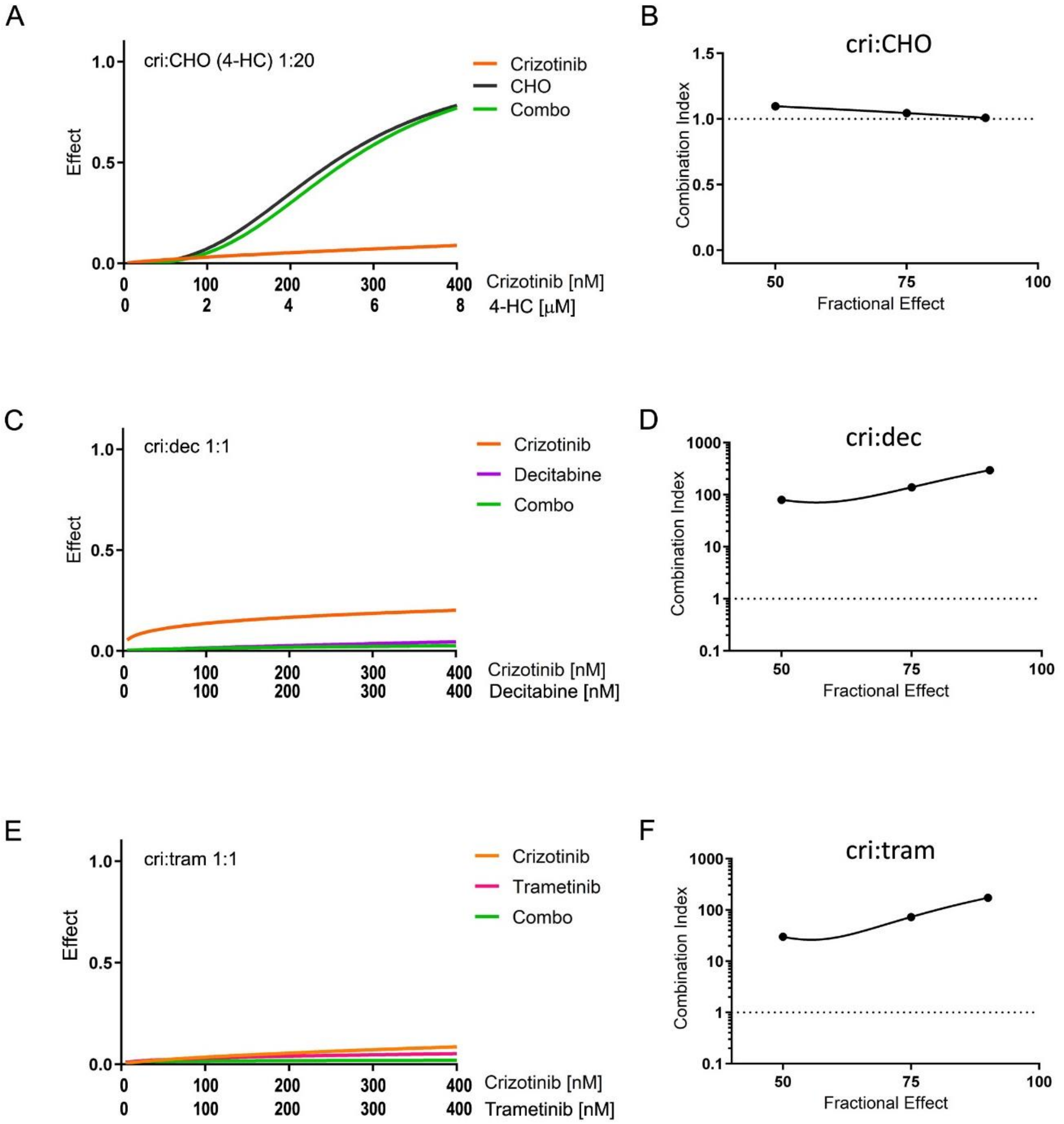

Figure S6. Synergism analysis in U937 cells. The cells were treated with vehicle, or single drugs, or combinations (**A,C,E**) Dose-effect curves and (**B,D,F**)combination index values were calculated as described in the legend of Figure 2. (**A,B**) Crizotinib+CHO; (**C,D**) Crizotinib+Decitabine; (**E,F**) Crizotinib+Trametinib.

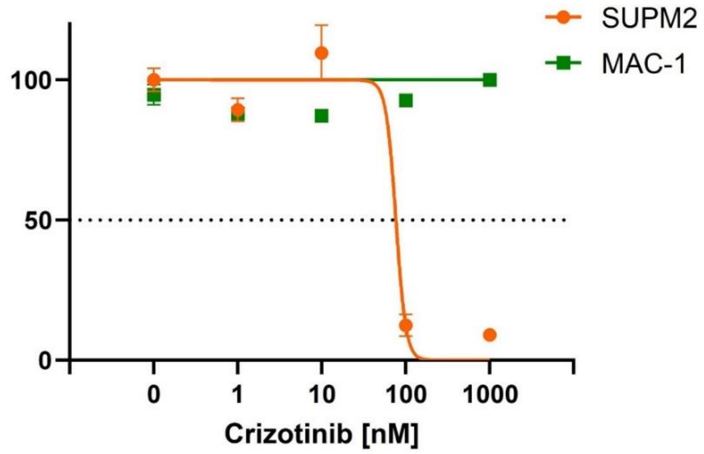

Figure S7. Insensitivity of ALK-negative ALCL cells to crizotinib. SUP-M2 (ALK+) and MAC1 (ALK-) cells were treated with increasing doses of crizotinib. MAC1 cells do not show any response to the drug up to 1 μM.

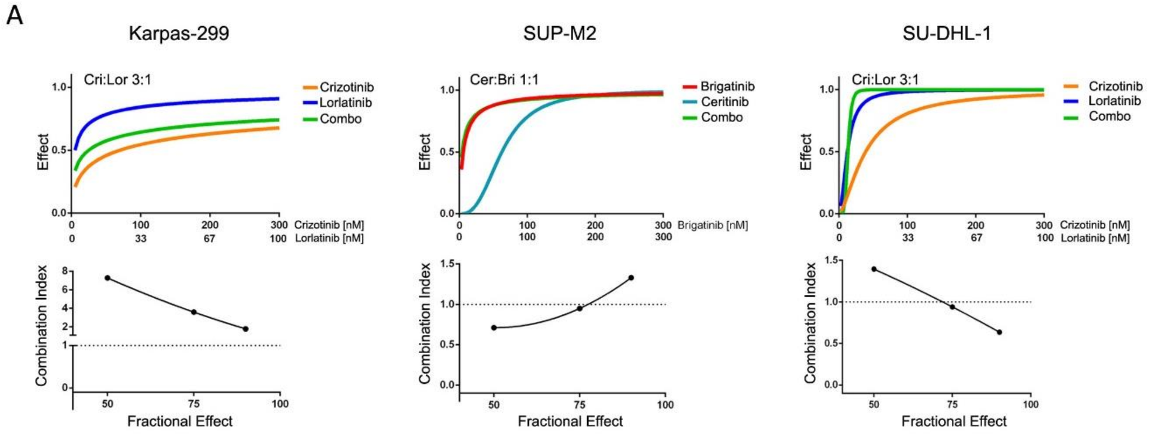

B

CRI:LOR	Karpas 299	3:1	7.286	3.594	1.782	4.221	Strong Antagonism
	Karpas 299	10:1	2.349	2.783	3.297	2.810	Antagonism
	Karpas 299	30:1	0.969	>10	>10	>10	Very Strong Antagonism
	SU-DHL-1	3:1	1.396	0.941	0.637	0.992	Nearly Additive
	SU-DHL-1	10:1	0.992	0.706	0.505	0.734	Moderate Synergism
	SU-DHL-1	30:1	1.558	1.102	0.783	1.148	Slight Antagonism
CER:BRI	Karpas 299	1:1	0.869	1.043	1.285	1.065	Nearly Additive
	Karpas 299	1:2	0.997	1.007	1.039	1.014	Nearly Additive
	Karpas 299	1:3	1.006	0.986	0.982	0.991	Nearly Additive
	SUP-M2	1:1	0.712	0.950	1.330	0.997	Nearly Additive
	SUP-M2	3:1	0.385	0.726	1.511	0.874	Slight Synergism
	SUP-M2	10:1	0.014	0.115	1.077	0.402	Synergism

Figure S8. Synergism analysis of two ALK inhibitors combo. (**A**) Examples of dose-effect curves and (**B**) the corresponding CI values obtained with two TKI combinations that were able to prevent drug resistance in long-term experiments. Green, yellow and red dots in panel B indicate synergism, additivity and antagonism, respectively.

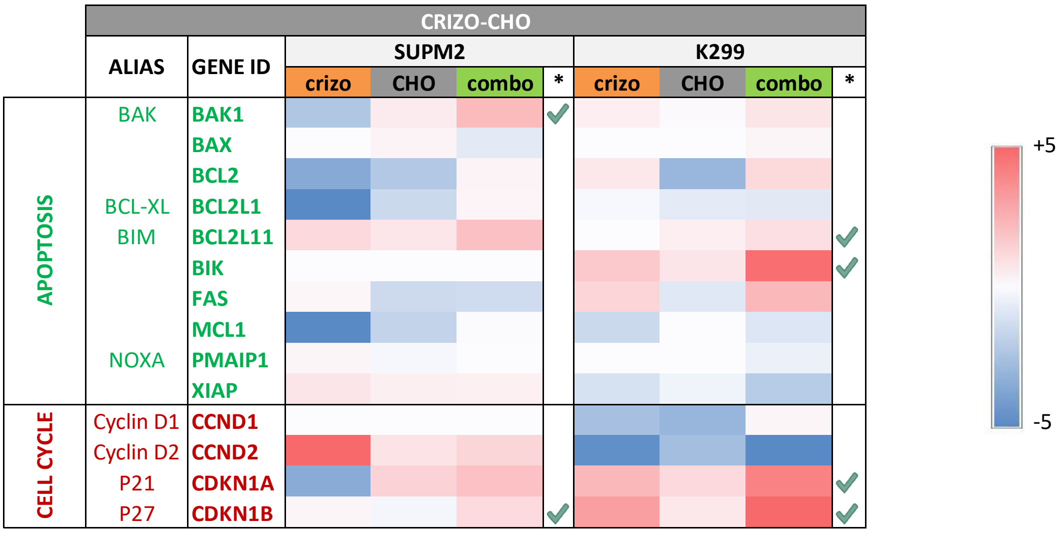

Figure S9. Heatmap of qPCR data obtained with crizotinib+CHO combination. The cells were treated for 72 hours, then total RNA was extracted and analyzed. Fold-change from DMSO-treated controls is reported. A tick in the right columns indicates significant changes in combo *vs* both singles.

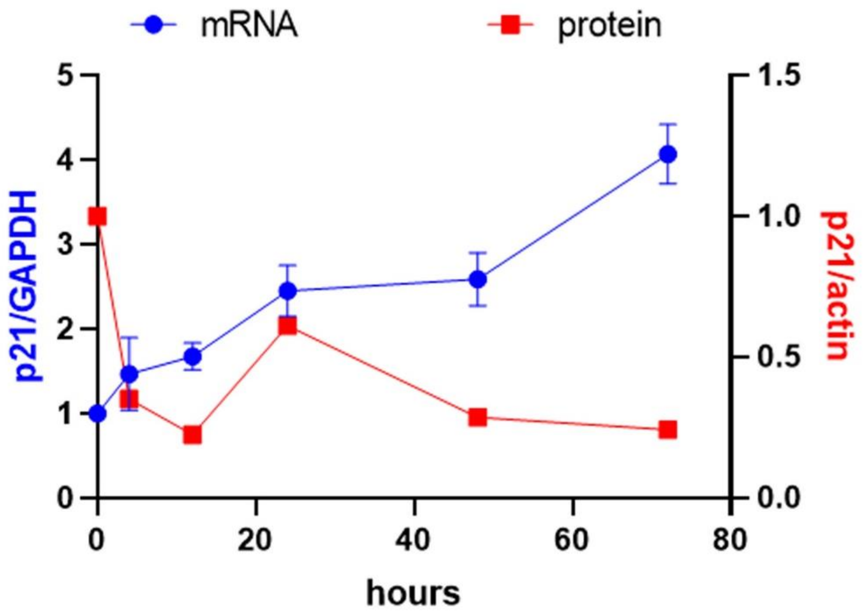

Figure S10. Time course of p21Waf1 expression in Karpas-299 cells treated with crizotinib+CHO. Cells were collected at 4, 12, 24, 48 and 72 hours from the start of treatment. RNA and proteins were extracted and analyzed. RNA expression was normalized to GAPDH; protein level was normalized to b-actin. Data are relative to time 0.

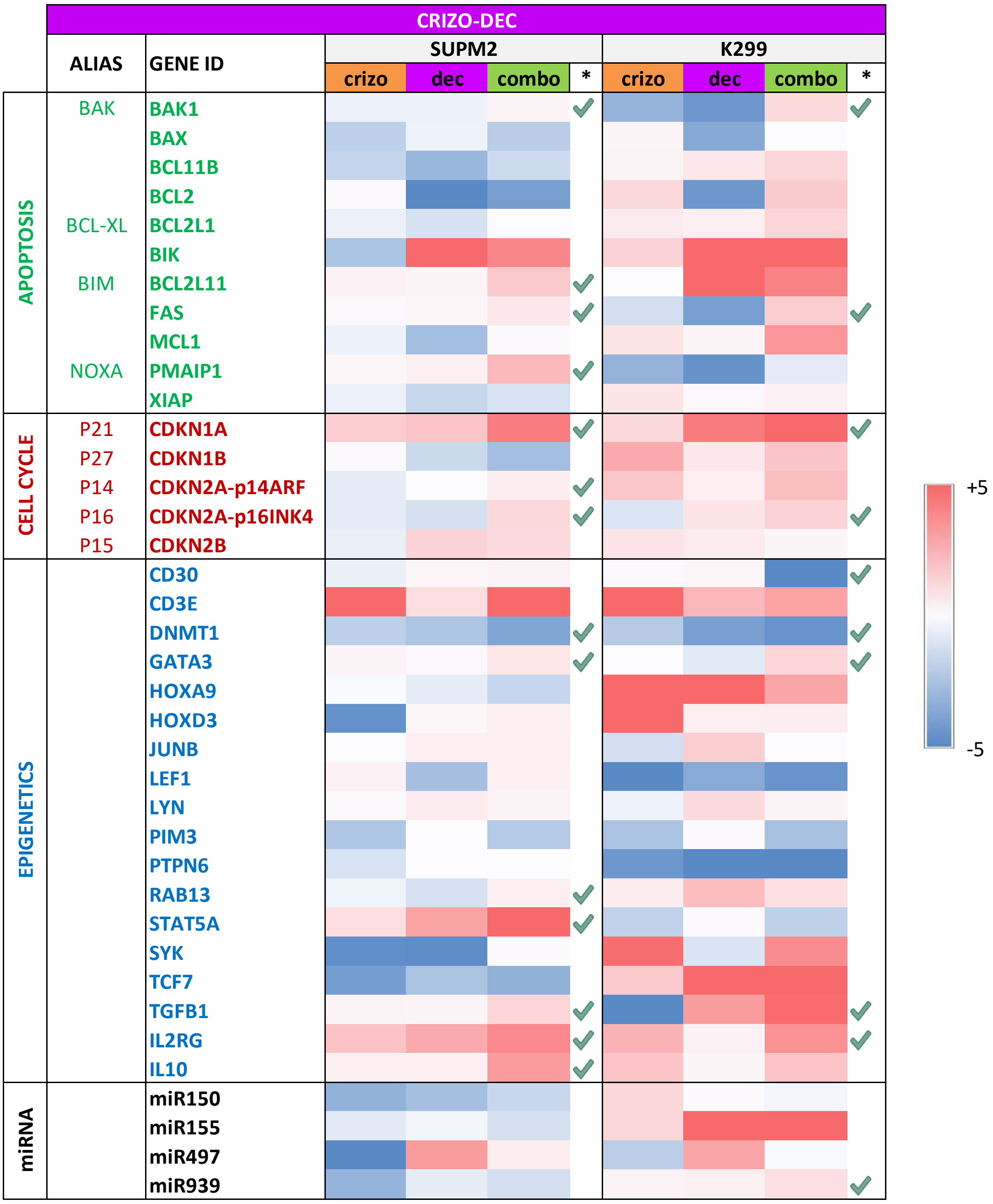

Figure S11. Heatmap of qPCR data obtained with crizotinib+decitabine combination. The cells were treated for 72 hours, then total RNA was extracted and analyzed. Fold-change from DMSO-treated controls is reported. A tick in the right columns indicates significant changes in combo vs both singles.

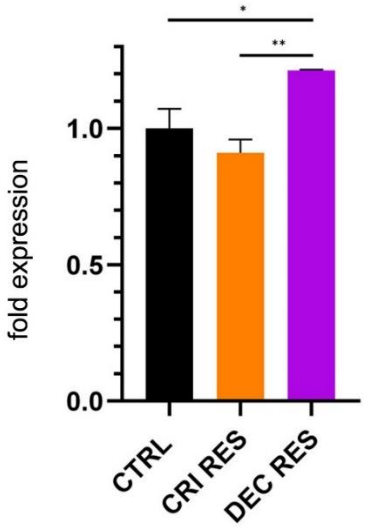

Figure S12. Real time PCR analysis of DNMT1 expression in SUP-M2 cells. CTRL = parental; CRI RES = resistant to crizotinib; DEC RES = resistant to decitabine.

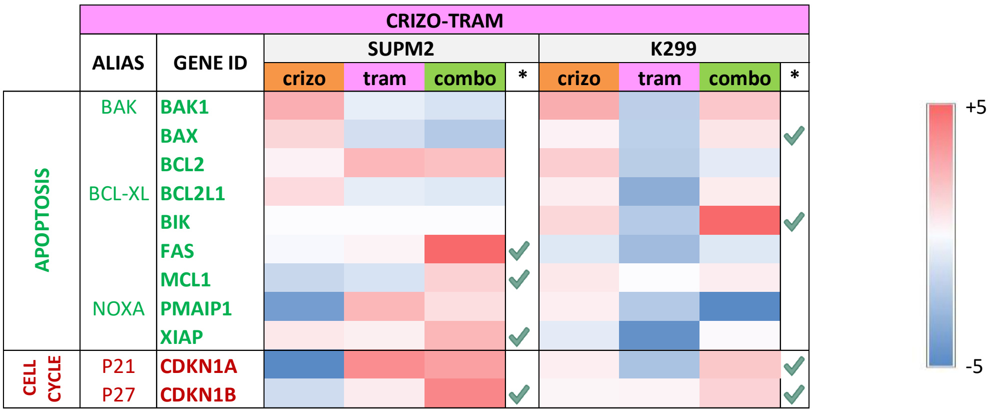

Figure S13. Heatmap of qPCR data obtained with crizotinib+trametinib combination. The cells were treated for 120 hours, then total RNA was extracted and analyzed. Fold-change from DMSO-treated controls is reported. A tick in the right columns indicates significant changes in combo *vs* both singles.

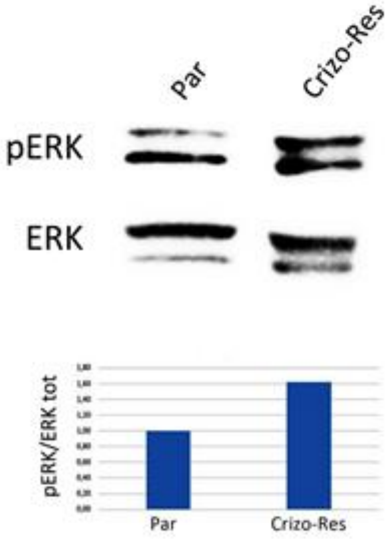

Figure S14. (**Top**) SUPM2 cells resistant to 120 nM crizotinib (Crizo-res) show 60% increase of ERK1/2 phosphorylation by Western blotting, compared to parental SUPM2 (Par). (**Bottom**) densitometry quantification of normalized pERK/total ERK signal.

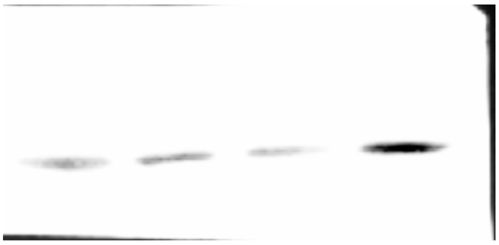

γH2AX

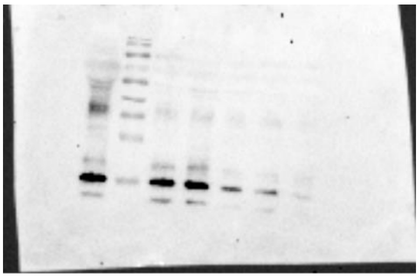

p21

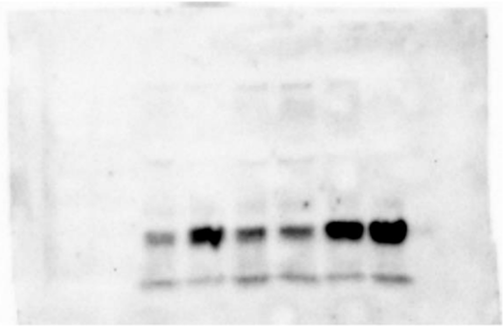

p27

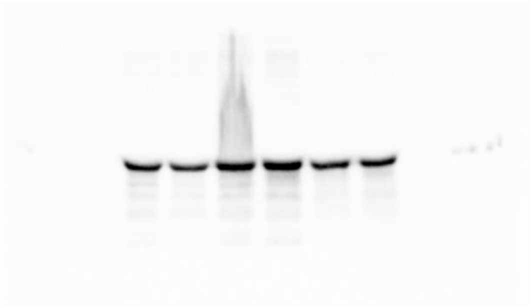

p53

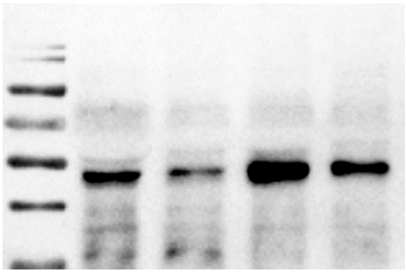

P-p53.

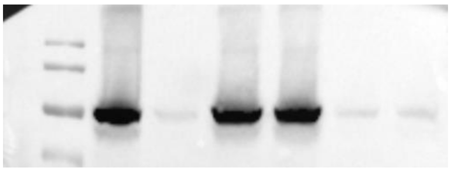

pSTAT3

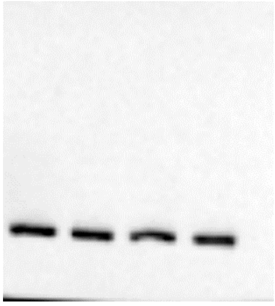

actin

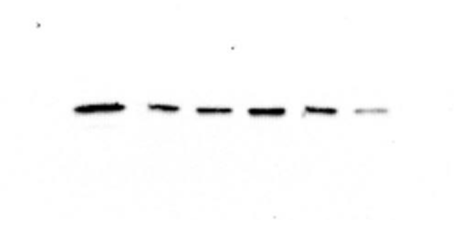

bcl2

Figure S15. Uncropped western blot figure for Figure 4E.

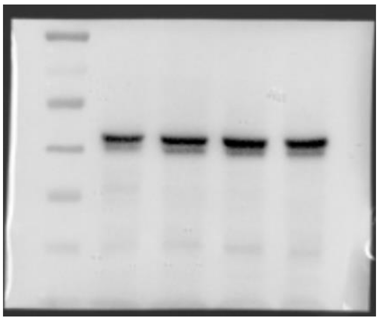

ERK

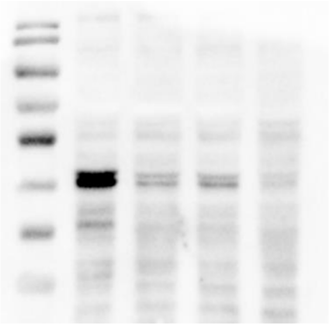

pERK

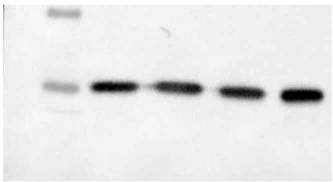

H3K4

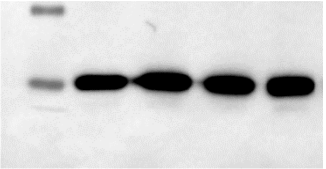

H3

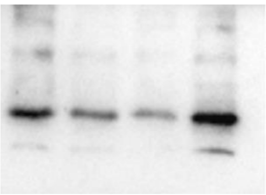

p16

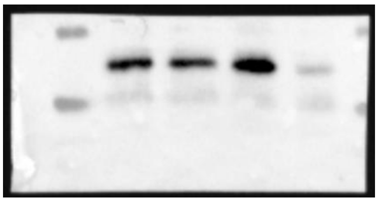

p21

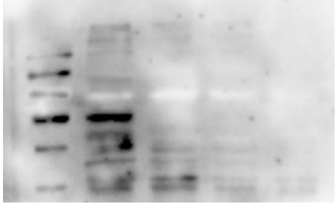

pJNK

p53

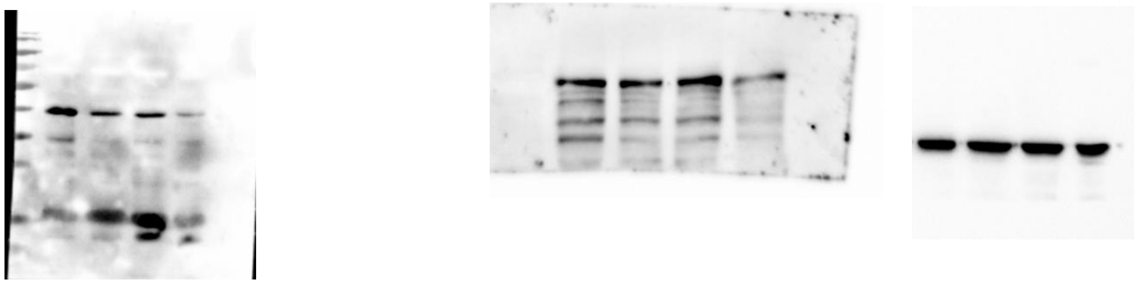

p-p38 **PLCγ** **actin**

Figure S16. Uncropped western blot figure for Figure 5G.

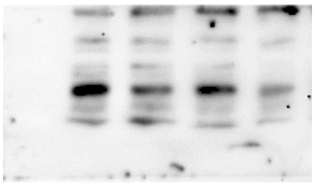
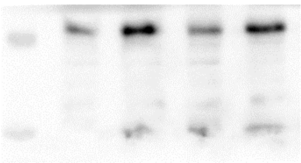

p21 **p27** **p53**

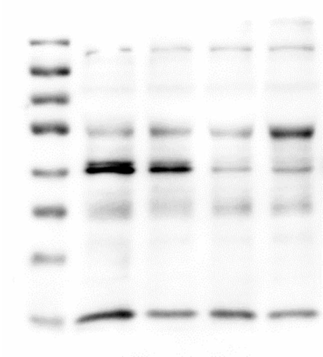
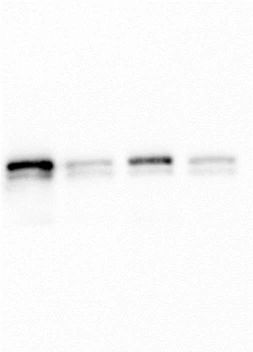
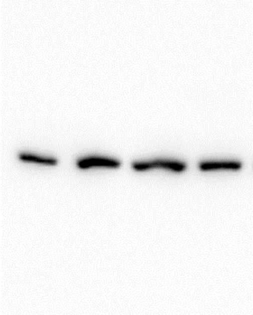

pERK **pSTAT3** **actin**

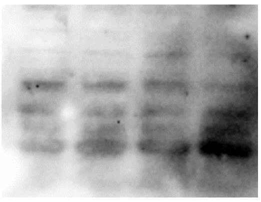
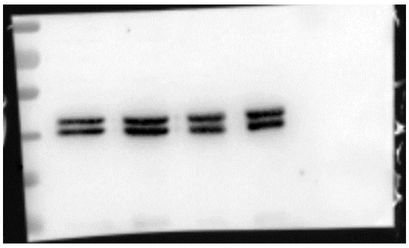
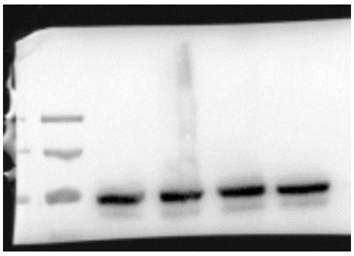

BIM **ERK** **STAT3**

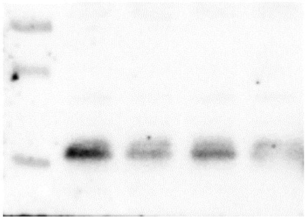

Survivin

Figure S17. Uncropped western blot figure for Figure 6A.

Table S1. Antibodies used in this study are listed.

ANTIBODY	MANUFACTURER	Cat#
Actin	Sigma	A2066
Bcl2	Cell Signaling Technology	2870

BIM	Sigma	B7929
Histone H3	Abcam	ab1791
Histone H3K4me3	Diagenode	C15410003
p16 INK4A (D7C1M)	Cell Signaling Technology	80772
p21 Waf1/Cip1 (12D1)	Cell Signaling Technology	2947
p27 Kip1 (C-19)	Santa Cruz Biotechnology	sc-528
p44/42 MAPK (Erk1/2)	Cell Signaling Technology	9102
p53 (DO-1)	Santa Cruz Biotechnology	sc-126
STAT3	Cell Signaling Technology	12640
Survivin	Santa Cruz Biotechnology	sc-17779
γH2AX (Ser139)	Millipore	07-164
Phospho-p38 MAPK (Thr 180/Tyr 182) (D3F9)	Cell Signaling Technology	4511
Phospho-p53 (Ser15) (16G8)	Cell Signaling Technology	9286
Phospho-STAT3 (Tyr 705)	Thermo Fisher Scientific	Ma5-15193
Phospho-p44/42 MAPK (Erk1/2) (Thr202/Tyr204)	Cell Signaling Technology	4370
Phospho-SAPK/ JNK (Thr183/ Tyr 185)	Cell Signaling Technology	9251

Table S2. Primers used in this study, not previously reported. Additional information can be found in refs. 19, 24.

**PRIMER***	**SEQUENCE (5'-3')**
CDKN2A-p14ARF-F	CTCGTGCTGATGCTACTGAGGA
CDKN2A-p16INK4a-F	GTAGAGGAGGTGCGGGCG
CDKN2A-R	GGTCGGCGCAGTTGGGCT
CDKN2B-p15INK4b-F	ACGGAGTCAACCGTTTCGGGAG
CDKN2B-p15INK4b-R	GGTCGGGTGAGAGTGGCAGG
DNMT1-F	CCATCAGGCATTCTACCA
DNMT1-R	CGTTCTCCTTGTCTTCTC
GATA3-F	ACCACAACCACACTCTGGAGGA
GATA3-R	TCGGTTTCTGGTCTGGATGCCT
IL2RG-R	GCCAGTCCCTTAGACACACC
IL2RG-F	ATTTCTGGCTGGAACGGACG
RAB13-F	GACATCTTGCTCAAGTCAGGAGG
RAB13-R	CAGGGAGCACTTGTTGGTGTTC
TGFB1-F	TACCTGAACCCGTGTTGCTCTC
TGFB1-R	GTTGCTGAGGTATCGCCAGGAA

*F = forward; R = reverse.
